# Consumer self-reported and testosterone responses to advertising of luxury goods in social context

**DOI:** 10.1007/s43039-021-00023-y

**Published:** 2021-04-21

**Authors:** Rumen Pozharliev, Willem Verbeke, Matteo De Angelis, Ruud Van Den Bos, Paolo Peverini

**Affiliations:** 1 LUISS Guido Carli, Viale Romania, 32, 00197 Rome, Italy; 2 Erasmus School of Economics, Erasmus University Rotterdam, P.O. Box 1738, 3000 DR Rotterdam, The Netherlands; 3 Radboud University, Comeniuslaan 4, 6525 HP Nijmegen, The Netherlands

**Keywords:** Luxury goods, Testosterone, Social status, Reward experience, Advertising

## Abstract

Despite the growing demand for luxury goods, there is limited understanding about how consumers respond to luxury-goods advertising and how viewing advertising in different social contexts affects these responses. This study investigates the link between luxury goods advertising and expected utility from a biological perspective by looking at males’ hormonal responses to advertising of luxury versus non-luxury branded goods. Using traditional and consumer neuroscience methods, we collected salivary testosterone data pre- and post-ad viewing. Self-reported scores on social and quality value of the brands were compared to salivary testosterone levels from participants placed in different social conditions (Alone versus Together with another person). The results show that higher post-viewing testosterone levels were associated with higher scores on quality, but not on social value and only for branded goods viewed in the Together condition, compared to the Alone condition. These results suggest that changes in testosterone levels reflect a rewarding experience or activate social competitiveness when male consumers appraise the quality but not the social value of the advertised luxury goods and that social context modulates this effect.

**Supplementary Information:**

The online version contains supplementary material available at 10.1007/s43039-021-00023-y.

## Introduction

The global demand for luxury goods including cars, jets, drinks and fashion has been constantly increasing for decades, resulting in an industry worth $285 billion in 2020 (Jones, 2020). Amid the COVID-19 crisis, the global luxury goods market is projected to reach a revised size of $403 billion by 2027, growing at a revised CAGR of 2.1% over the period 2021–2027 (Wood, 2020). Despite the COVID-19 global pandemic, sales of luxury cars will continue to dominate the market for luxury goods, growing 5% at constant exchange rates over the period 2021–2027 (Fortune Business Insights, 2020). As a result, luxury-goods marketing has generated much interest from both academic and practitioners. Prior marketing literature on luxury goods consumption reports a diverse range of motivations for consuming luxury products including, for example, signaling of social status (e.g., Han et al. 2010), the pursuit of pleasure and rewarding experience (e.g., Dubois et al., 2005), and the need for uniqueness (e.g., Zhan & He, 2012).

An analysis of the motivations that typically drive consumers’ decision to buy luxury goods suggests that consumers see in many cases the attainment of luxury goods as the fulfillment of a dream. Recent research on luxury consumption has highlighted that luxury brands’ communication is typically oriented to instill dream and aspiration in consumers (Amatulli et al., 2020), via the employment of different tactics, such as, for example, the use of imagery rather than text (Amatulli et al., 2018), the use of celebrities (Erdogan & Drollinger, 2008) and the use of hedonic rather than utilitarian advertising appeals in textual advertisements (Amatulli et al., 2020). However, one important factor that prior studies on luxury goods communication seem to have overlooked is the context in which consumers are exposed to luxury goods communication and how it can affect consumers’ perceptions of advertised goods. Building on prior literature that stressed out the importance of studying consumer responses to advertising in social contexts which resemble the contextual environment in which real-life advertising is typically experienced (Pozharliev et al., 2017; Puntoni & Tavassoli, 2007), we address this gap by exploring whether and how the presence of another person in the environment in which a consumer is exposed to an advertisement affects consumer responses to luxury-goods advertising. In particular, we investigate how consumer perception of the *social value* defined as “the utility derived from the product’s ability to enhance social self-concept” and *quality value* defined as “the utility derived from the perceived quality and expected performance of the product” (Sweeney & Soutar, 2001 p. 211) affects their responses to luxury-goods advertising (Amatulli et al., 2020; Han et al., 2010; Vigneron & Johnson, 2004; Wang & Griskevicius, 2013). Gaining a better understanding on how contextual variables (e.g., the. social context) affect consumer responses to advertising of luxury goods is of critical importance for companies and brands that are looking to thoroughly assess the impact of their marketing communication. In particular, our study builds on previous marketing research that acknowledges that consumer behavior to branded goods (luxury and non-luxury) is affected by other people—friends, family, strangers or salespeople (Jayasinghe and Ritson 2012; Kurt et al. 2011; White & Dahl, 2006). Marketing is a social activity, and much work has already alluded to this dimension of consumer responses to marketing communication (Argo et al., 2005; White & Argo, 2011). Previous research suggests that consumer attend differently to marketing related information when viewed alone than in the co-presence of others (Pozharliev et al., 2015).

Moreover, previous research on consumer responses to advertising of luxury goods relied exclusively on self-reports that largely depend on the willingness and the ability of consumers to describe their responses to advertising of luxury goods (Amatulli et al., 2020; Chu et al., 2019; Kwon et al., 2016; Phillips & McQuarrie, 2011). However, many unconscious processes may have a considerable impact on consumer responses to marketing stimuli (Plassmann et al., 2012; Verbeke et al., 2014). People sometimes have subtle feelings of knowing what they have experienced in relation to advertising exposure, although they may be unable to retrieve explicit information from their memory and express it in words (Gordon et al., 2018). Likewise, consumers’ emotional responses to advertising are complex and often include unconscious processes which are difficult to reproduce verbally (Shaw & Bagozzi, 2018). In other words, previous studies have largely neglected the physiological processes that might affect consumer responses to luxury goods advertising (Venkatraman et al., 2015). Indeed, neuroscience has quite recently been used in marketing for investigating consumer responses to advertising (Pozharliev et al., 2017), and the philosophical and methodological grounding of these methods have recently received considerable scrutiny (Bagozzi & Lee, 2019). Consumer neuroscience, which consists in the use of neurophysiological and biological measures of consumers’ reactions, offers safe and noninvasive access to consumers’ unconscious processes and physiological responses to such stimuli as print and television ads, and is therefore often used to complement traditional self-reported measures (Plassmann et al., 2012; Shaw & Bagozzi, 2018; Venkatraman et al., 2015). In particular, previous research suggests that changes in hormonal levels might be a reliable and relevant consumer neuroscience method to study consumer emotional responses to advertising of luxury goods (Nave et al., 2018; Pozharliev et al., 2017). Specifically, changes in testosterone levels (hereafter, T levels) have been associated to fluctuations in consumers’ status that were triggered by act of conscious consumption (Saad & Vongas, 2009), and social status have been frequently reported as one of the main drivers of luxury goods consumption (Han et al., 2010). However, to the best of our knowledge, no prior research has studied consumer hormonal responses (e.g., T levels) to advertising of luxury goods. Thus, we extend previous consumer neuroscience research applied to the study of consumer responses to marketing communication by investigating the association between luxury value appraisal and salivary T levels in male consumers viewing advertising of luxury versus non-luxury branded goods (Plassmann et al., 2012; Pozharliev et al., 2015). Studying the association between hormones and luxury value appraisal provides a window, complementary to self-reports, into the physiological processes that influence consumer responses to advertising. In turn, these insights may help marketing professionals understand the biological processes that influence consumer responses to luxury-goods advertising, which could help them improve the allocation of marketing communication resources to better match customers’ motives and preferences and eventually translate into sales (Lee et al., 2017; Venkatraman et al., 2012). Specifically, our results show higher post-viewing testosterone levels were associated with higher scores on quality value, but not on social value.

Overall, therefore, our study is the first, to the best of authors’ knowledge, to study consumer hormonal responses to advertising of luxury goods in a social context. Our results indicate that social context enhances consumer hormonal responses to advertising of luxury goods.

Finally, our research might also offer practical implications for marketing communication managers looking for new ways to enhance the impact of their marketing communication, and in particular for those managers interested in empirical evidence supporting their decision to invest in luxury goods advertising. First, regardless of the level of perceived quality and social value of the luxury good, marketers should try to create social platforms on which consumers can experience the luxury goods advertising intensively. Second, advertising of luxury goods should leverage not only the social, but also the quality value of the advertised products. Finally, marketers should incorporate consumer neuroscience methods to better understand and predict consumer responses to their marketing communication.

The paper is organized as follows. First, we discuss the differences between luxury and non-luxury branded goods in terms of social and quality value. Second, we report past empirical evidence on the association between salivary testosterone, social context and consumer responses to marketing stimuli. Third, we develop our hypotheses on whether exposure to ads of luxury versus non-luxury branded goods is reflected differently in the post-viewing T levels and whether the presence of another person modulates these differences. Next, we present our materials, including the experimental setup, hormonal and self-reported data collection, statistical procedures, and report the results. Finally, we discuss the theoretical and practical implications of our findings and provide suggestions for future research.

## Conceptual development

Understanding what a customer values in products is of crucial importance to marketing managers seeking to achieve competitive advantage. Past marketing research on product value has largely focused on the trade-off between quality and price, thus outlining the importance of functional value in consumer behavior. For instance, Zeithaml (1988, p. 14) defines perceived value as a “consumer’s overall assessment of the utility of a product based on perceptions of what is received and what is given.” Other authors argue that this definition is too narrow, suggesting that other constructs such as *social or quality* dimensions have an equal if not a larger impact on consumers’ overall perceived value (Sweeney & Soutar, 2001; Wang & Griskevicius, 2013).

### Social value

Psychological research suggests that the need for social status is a main driver of the desire for luxury goods (Drèze & Nunez, 2009; Rucker & Galinsky, 2008). Evolutionary psychology proposes that human desire for luxury goods stems, in part, from the universal tendency to signal traits that enhance social status and for some persons boost the chances of finding a mate who can assure healthy offspring (Miller, 2009). In contrast, low status comes with undesirable consequences, such as less access to resources (Lin & Dumin, 1986). Traditionally described as goods which use, display, or bring the owner social value in terms of conspicuousness and prestige in social networks, luxury goods are thus often spontaneously desired by consumers (Rucker & Galinsky, 2008; Wang & Griskevicius, 2013). The desire might show up in how they imagine other people would view them if they possessed a luxury good (Wiedmann et al., 2007). For instance, a Jaguar fan characterized his driving experience as one that generates high social value: “I love the way that I catch people admiring the XJ-S when I blast past them, and the way that people often give me right of way in traffic and then watch the car as it goes by” (Atwal & Williams, 2009, p. 45). Sweeney and Soutar (2001, p. 211) define social value as “the utility derived from the product’s ability to enhance social self-concept.” Note that the authors use the term “ability to enhance social self-concept”, reflecting an expectation, wanting or desire for self-enhancement. Self-presentation motives often drive consumers to prefer brands that can satisfy their desire to resemble the typical brand user and avoid brands associated with undesirable reference groups (Escalas & Bettman, 2005; White & Dahl, 2006). Consumer preference for a luxury car rather than a non-luxury car could indicate a person’s desire to yield social benefits such as facilitated social interaction with people high in social status.

### Quality value

Gentry et al. (2001) suggest that consumers may obtain more value from luxury goods because they associate them with superior quality, reassurance, and performance compared to non-luxury goods. Specifically, consumers influenced by the quality dimension of luxury may have learned through observation and/or experience that luxury goods have distinctive physical attributes such as superior technology, innovative design, comfort, reliability, artisanship, and that they will last longer compared to non-luxury goods (Sweeney & Soutar, 2001). For instance, speed, acceleration and the exterior design of a sports car or the exclusive interior, quality materials and comfort of a luxury limousine all reflect the perception of quality. This suggestion resonates with the general belief that luxury brands offer first-class product quality and performance compared to non-luxury brands (Aaker, 2009; Dubois & Laurent, 1994; Vigneron & Johnson, 2004). Thus, when consumers see luxury goods, they expect them to have high quality. Previous research indicates that the social and quality dimensions of luxury are highly correlated (Sweeney & Soutar, 2001). Benjamin and Podolny (1999) show that status comes with higher expectations of quality. Despite this high correlation, we expect that both dimensions contribute distinctively to the perception of luxury (Vigneron & Johnson, 2004).

Luxury brands have been conceived as creating specific expectations, in that they can enable a gain in social status or provide comfort (Vigneron & Johnson, 2004). Such expectations come from learning experiences, ranging from a person’s own experience of the brand, vicarious learning from other consumers or opinion leaders and, of course, marketing tools such as branding and pricing (Lee & Watkins, 2016; Plassmann & Wager, 2014). Importantly, these expectations evoke expected utility, an activity that occurs when someone is watching or paying attention to branded goods, and as such it evokes expectations and wanting (Berridge & Robinson, 2016). In practical terms, it is not surprising that many consumers confess that shopping without consuming or buying branded goods (e.g. browsing, window shopping) is a nice pastime because it fulfills their desire to possess these goods, even if only in imagination (Arnold & Reynolds, 2003; Brakus et al., 2009). Plassmann and Wager (2014) label this process the “placebo effect of luxury goods on the consumer”, meaning that luxury goods are expected to have higher quality or are expected to provide consumers with social benefits, which might evoke dreams of how others might regard them if they obtained the associated high status.

In summary, luxury branded goods offer superior quality, reassurance, and performance compared to non-luxury goods (Dubois & Laurent, 1994; Gentry et al., 2001; Sweeney & Soutar, 2001). They have better physical characteristics, such as faster speed, quality workmanship, and innovative technology. In addition, luxury brands are associated with social benefits (Wiedmann et al., 2007). They can signal high social status, success, and affiliation to prominent reference groups (Escalas & Bettman, 2005; Vigneron & Johnson, 2004). Thus, we hypothesize:

#### H_1_

Luxury branded goods shown in a video advertisement will score higher on social and quality value than non-luxury branded goods.

### Testosterone, social behavior and social status

Testosterone responses provide a reliable and long-lasting measure of consumer responses which perfectly fits the specific purpose of this study, aimed at understanding the overall response of consumers to the advertising of luxury goods (Carré & Putnam, 2010; Pound et al., 2009; Saad & Vongas, 2009; Van der Meij et al., 2012). Testosterone is a steroid hormone secreted primarily by the testicles of males and the ovaries of females. The adrenal cortex also secretes it in both sexes (Mazur & Booth, 1998). Approximately 97% is inactive testosterone due to its bonding to three major proteins in blood. The remaining 3% circulates freely and produces most of the observable behavioral effects (Saad & Vongas, 2009). This free form is found in saliva and thus hormonal research on T levels uses salivary rather than blood or urine measurements (Eisenegger et al., 2011). Salivary testosterone has proven to be a reliable noninvasive biomarker in clinical, social and economic research (Coates & Herbert, 2008; Newman et al., 2005). Previous studies on salivary testosterone point to a positive association between testosterone, social status and status-striving behavior (Eisenegger et al., 2011; Newman et al., 2005) and social status have been frequently reported as one of the main drivers of luxury goods consumption (Han et al., 2010), which makes it an appropriate tool for studying consumer responses to advertising of luxury goods. For instance, enhanced T levels were found in relation to higher levels of energy, status-seeking and competitive behavior (Carré & Putnam, 2010).

Two main overlapping explanations about the rise in T levels have been proposed. The biosocial model of status, suggesting that people seek to defend their status, proposes that T levels rise when people either win a contest or are about to defend their earned status (Mazur & Booth, 1998). The challenge hypothesis proposes that when people’s status is challenged they will produce higher T levels (Archer, 2006). Prime examples come from sports. For instance, soccer players entering a stadium where they have previously won a match, or when playing on home ground, automatically experience a peak in testosterone because they expect to win in these specific contexts (Geniole et al., 2017; Gleason et al., 2009). Hence, T levels are context-sensitive in that people become attached to or develop expectations of their position in these contexts (Salvador & Costa, 2009). In addition, personality traits also affect T levels. For instance, people with a strong need for power have high T levels on encountering a challenger or merely on being primed with a status cue (Schultheiss et al., 1999).

Marketing studies on hormones are scarce. In one of the few studies, Saad and Vongas (2009) showed that driving a luxury sports car versus a family sedan produces higher T levels compared to baseline T levels. In other words, luxury goods affect the endocrine system of consumers. While Saad and Vongas’ (2009) research reflects experienced value, it is likely that merely seeing or talking about luxury branded goods evokes expectancy or wanting and activates the testosterone system, hence triggering status goals. For instance, Carré and Putnam (2010) found that watching a previous victory enhances T levels, whereas watching a neutral video did not affect T levels. Van der Meij et al. (2012) reported enhanced T levels while watching a soccer match. In a placebo-controlled experiment Nave et al. (2018) found that administering testosterone increases men’s preference for status brands. Finally, previous research indicates that the different dimensions of luxury are highly correlated (Sweeney & Soutar, 2001; Vigneron & Johnson, 2004). We expect that the testosterone response to watching ads of luxury branded goods will be associated with both utility dimensions, because social and quality value are both scarce and provide comfort that people seek and defend, leading to activation in terms of a rise in T level (Vigneron & Johnson, 2004). Thus, we hypothesize that:

#### H_2_

Participants’ reported scores on quality and social value for branded goods viewed in an advertising will relate positively to their post-viewing T levels.

### Testosterone and social context

T levels change in social situations (Josephs et al., 2011). Even though animal research confirmed the importance of testosterone as a “social hormone” decades ago, the study of its impact on human behavior in social interactions and social context has only begun recently (Eisenegger et al., 2011). Consumer status motivation is a natural response because of the rewarding benefits associated with high status. Importantly, humans are capable of creating status domains for themselves without having to possess an absolute hierarchical position because status can be derived in different social contexts, for instance, being an early adopter of green products, a well-known artist or a well-known socially responsible person (Griskevicius et al., 2010).

Due to the complexity of social contexts and their diverse reciprocal effects on T levels, most advanced research has administered testosterone in the laboratory. These studies show that testosterone administration comes with lower levels of threat or anxiety when participants view pictures of human faces of negative valence in the Alone condition (Eisenegger et al., 2011; van Honk et al., 2005). Note that in the laboratory, participants are isolated from a concrete social context with other conspecifics. Pozharliev et al. (2017) discussed the way social context may affect consumers’ neurophysiological responses to marketing stimuli by providing a framework of interaction between social and cognitive processes. Pozharliev et al. (2015) found that social context modulates consumer’s neurophysiological responses to luxury branded goods. Specifically, the authors reported enhanced attention allocation to luxury branded goods viewed in the mere presence of others compared to being alone.

Previous research indicates that the social context in which a visual stimulus is experienced modulates a range of behavioral, cognitive and neurophysiological responses (Akitsuki & Decety, 2009; Pozharliev et al., 2017). For instance, social context influences viewers’ attention allocation to TV ads (Moorman et al., 2012), emotional responses (Fisher & Dubé, 2005), memory (Puntoni & Tavassoli, 2007) as well as preference and purchase behavior (Ariely & Levav, 2000; Luo, 2005). Past literature indicates that context is an important variable that can affect testosterone responses to a socially silent stimulus (Casto & Edwards, 2016). Previous studies indicate that social context modulates neurophysiological responses to luxury branded goods. For instance, Pozharliev et al. (2015) found increased brain activity in attention-related areas when participants viewed pictures of luxury branded goods together with another person compared to when they viewed them alone. Thus, we hypothesize that:

#### H_3_

The social context will moderate the positive relation between participants’ reported scores on social and quality value for branded goods viewed in an advertising and their post-viewing T levels.

Given that circulating levels of testosterone are higher in male than female subjects, the effects of levels of testosterone on reward processing have been studied either by a correlational approach using circulating levels of testosterone in males, i.e., making use of natural variation, or by administering testosterone to females to increase current levels (Carré & Putnam, 2010; Hermans et al., 2010). Here we study male subjects using a correlational approach as we assume that competitive social status is more relevant to male subjects than female subjects.

## Research design and methodology

### Participants

Ninety-six male (age M = 22.50 years, SD = 2.24) healthy undergraduate students from an EU university participated in this study. Participants enrolled in the experiment in exchange for course credit. All participants had normal or corrected-to-normal vision. The study was approved by the university’s Ethics Committee. Informed consent was obtained from each participant at the beginning of the experiment.

### Materials

The stimuli used consisted of 14 videos (ads) showing various car brands. The ads were selected by two male professional marketers who received payment for the task. They chose seven ads showing luxury cars and seven showing non-luxury branded cars. Web Appendix A provides details on the two sets of ads. In a pretest, 40 male undergraduates were asked to classify the 14 ads by indicating which branded cars they perceived as luxury and as non-luxury. A branded car was luxury or non-luxury if at least 90% of the participants rated it as such. The results of the pretest matched with the two sets (luxury and non-luxury) provided by the three male professionals. Each set of seven ads was approximately 13 min long.

### Questionnaires

We used the well-established scale of perceived value of branded products (PERVAL) to measure participants’ appraisal of social and quality utility at the brand level (Sweeney & Soutar, 2001). Questions 1–6 assessed quality utility and questions 7–10 assessed social utility of branded goods (for further information, see Web Appendix B). For instance, Sweeney and Soutar (2001, p. 211) describe the quality value dimension as “the utility derived from the perceived quality and expected performance of the product.”

### Procedure

Upon arrival at the lab, participants were given time to get acquainted with the experimenter. They were informed about the coming events, the questionnaire they would be asked to fill in and the number of saliva samples that would be collected. They were left alone in a separate experimental room for 15 min, where they were asked to carefully read and then sign the informed consent form and to relax for the remaining time. Hormones are slow moving, therefore, these 15 min of rest were needed in order to make people relax and bring their hormonal levels to their normal state. After 15 min they were asked to provide the first saliva sample (T1) which would later be used in the analysis as a pre-experimental baseline measure. Next, participants were moved to another experimental room where the fully computerized stimulus presentation task was conducted. They were randomly assigned to one of the two experimental conditions (Alone or Together) and to one of the two ad sets (luxury or non-luxury). In the Alone condition, a single participant sat in a comfortable chair approximately 100 cm from a 40-by-30 cm Iiyama PC computer screen set at eye level. In the Together condition, the two participants sat beside each other, both facing the computer screen. In all conditions, the leader of the experiment left the room, ensuring that his presence did not affect the findings.

**Fig. 1:**
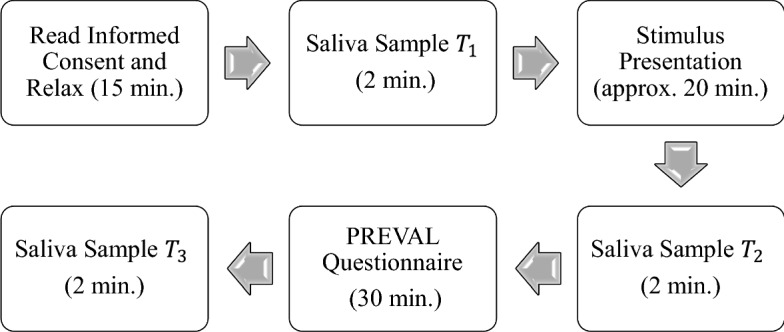
Experimental design

In both conditions, participants viewed a succession of seven ads displayed centrally on the computer screen using E-prime presentation software. The ads were presented in random order, and each ad was viewed once only. Each ad was followed by stimulus-free period of 20 s. Participants were instructed to watch the visual stimuli without making any overt responses. Participants were informed that during the stimulus-free period they were allowed to verbally express their opinion about the brand presented in the ad. In both (Alone and Together) conditions they were asked to verbally express their opinion about the brand in the ad using a static microphone (Logitech® Dialog-320). They were not given any specific indications or questions to answer, but were left to express their thoughts and feelings spontaneously. Everything the participants said about the brands in the ads was recorded, transcribed and analyzed by two independent linguistic specialists. Immediately post-viewing, participants returned to the initial experimental room where they were asked to provide the second saliva sample (T2) which would later be used in the analysis as a hormonal response to the visual stimuli. Next, they were asked to fill in the PERVAL questionnaire about the branded goods (luxury or non-luxury) in the ads they have been exposed to (Fig. 1). They were given 30 min to complete the questionnaire and to relax. Afterwards, participants were asked to provide the final saliva sample (T3) which would later be used in the analysis as a post-experimental baseline measure.

### Saliva collection and analysis

Collecting pre- and post-task hormonal data is a benchmark in the neuroscience research that looks at changes in hormonal levels in relation to external stimuli (Carré & Putnam, 2010; Pound et al., 2009; Saad & Vongas, 2009; Van der Meij et al. 2012). In the nervous system, signals travel very quickly, leading to instantaneous responses. However, within the endocrine system, signals move slowly but last longer. Hormones are usually slower acting. Previous research suggests that observable change in the level of the hormonal responses can be detected between 10 and 20 min after stimulus onset and that it takes around 20 min for the hormonal levels to return back to baseline (Carré & Putnam, 2010; Pound et al., 2009; Saad & Vongas, 2009; Van der Meij et al. 2012). Thus, the pre-task (T1) testosterone level represents the pre-experimental baseline measure and the post-task (T2) testosterone level represents the change of the hormonal response related to the task. In line with previous testosterone studies participants were asked to provide (30 min after the T2) a final saliva sample (T3) which would serve as post-experimental baseline measure (Carré & Putnam, 2010; Pound et al., 2009; Saad & Vongas, 2009; Van der Meij et al. 2012).

Saliva samples were collected before ad viewing (pre-experiment baseline measure), immediately post-viewing and 30 min after that (post-experimental baseline measure) using a commercially available collection device (Salivette®, Sarstedt, Germany), (see Fig. 1). Participants were left in private to collect their samples. The samples were stored at − 20 °C until assayed. The first author placed all the saliva samples in a cooler and took them to a laboratory at University Medical Central (UMC) in Utrecht, where they were analyzed (see below). Participants were asked to avoid food, smoking, alcohol consumption, visual and auditory sexual stimuli and not to brush their teeth in the three hours before the assessment. They were seated in the laboratory for 15 min prior to providing the pre-experiment baseline sample. To minimize variability due to diurnal fluctuations in circulating testosterone, all experimental sessions took place between 13:00 and 17:00 and for the specimens collected for this experiment there were no significant associations between testosterone concentrations and specimen production times. All participants were informed that saliva samples would be collected before and after the experiment. They were fully debriefed at the end of the experiment.

T levels in saliva samples were measured at the UMC Utrecht (UMC Utrecht; LKCH—Speciëel Laboratorium). Samples were measured in duplicate using an in-house competitive radio-immunoassay employing a polyclonal anti-testosterone-antibody (Dr. Pratt AZG 3290). [1,2,6,7-^3^H]-Testosterone (NET370250UC, PerkinElmer) was used as a tracer following chromatographic verification of its purity. The lower limit of detection was 10 pmol/L. Inter-assay variation was 9.1, 4.3 and 5.6% at 95, 200 and 440 pmol/L, respectively (n = 12, LKCH SL protocol 1610). Intra-assay variation was 7.2–2.5% at 38–92 pmol/L respectively (n = 10). Male reference values were for morning: 190–480 pmol/L; and evening: 83–240 pmol/L. Seven participants failed to deliver enough saliva for T-level analysis. This did not significantly affect the distribution of subjects across the different conditions. Thus, the final sample consisted of 89 subjects: 23 in Alone/Non-luxury, 21 in Together/Non-luxury, 22 in Alone/Luxury, and 23 in Together/Luxury.

### Statistical analysis

To test our hypotheses, we conducted a multivariate analysis of variance (ANCOVA) with Condition (Alone, Together), Social, Quality, and the interaction between Condition and the two luxury dimensions (Condition * Social and Condition * Quality) as independent variables and the T-level difference as a dependent variable. We controlled for multivariate normal distribution with Mauchly’s test of sphericity and applied the Greenhouse–Geisser correction, when appropriate. We considered a p-value less than 5% significant. Significant interaction effects were followed by paired sample t-tests. We implemented Bonferroni correction to adjust for multiple comparisons and analyzed statistics with the IBM SPSS 13.0 software.

## Results

### Perceived value of branded products (PERVAL)

To test ${\mathrm{H}}_{1}$ we computed an independent samples *t*-test with the product brand (Luxury, Non-luxury) as independent variable and the two PERVAL variables (Social and Quality) as dependent variables. As expected, participants evaluated the luxury brand products significantly differently from the non-luxury brand products on both social dimension (M_luxury_ = 5.23, SD_luxury_ = 0.84 vs. M_non-luxury_ = 3.43, SD_non-luxury_ = 0.83; t (87) =  − 10.18, *p* = 0.000) and quality dimension (M_luxury_ = 5.51, SD_luxury_ = 0.88 vs. M_non-luxury_ = 4.60, SD_non-luxury_ = 0.68; t (87) = − 5.49, *p* = 0.000).

### Hormonal results

The hypotheses assume an increase or decrease in T levels in relation to an individual’s baseline T. Therefore, we compared the participant’s two baseline samples (at T1 and at T3) to evaluate which of the two most accurately indicated their true baseline T levels. The initial contact with the laboratory environment, the researcher, as well as the unknown test condition ahead can collectively act as stressors or evoke anticipatory excitement leading to an increase in participants’ T levels (Saad & Vongas, 2009). As such, the final sample might be a better indicator of an individual’s true baseline than the first (Saad & Vongas, 2009). To test this assumption, we performed a paired *t*-test comparing the two baselines that revealed that T levels of sample T1 (M_sample T1_ = 234.94 pmol/L, SD = 63.83 pmol/L; range 117–451 pmol/L) were significantly higher than those from sample T3 (M_sample T3_ = 224.72 pmol/L, SD = 59.19 pmol/L; range 112–402 pmol/L), t (87) = 3.30, *p* = 0.001). Our results conform to Saad and Vongas (2009). Thus, to test our hypotheses, we used the baseline values from sample T3 as a reference value for determining changes in T level after viewing the ad (T levels of sample 2). It should be noted that the testosterone levels are within the range indicated above.

### Changes in T levels

Table 1 reports the descriptives and intercorrelations of our variables of interest. As expected, the perceived values (Social and Quality) of the branded goods correlated significantly with brand manipulation. The other variables (Condition, T response) were not significantly correlated with any other variable on a bivariate level.

**Table 1:** Correlations among variables of interest

	*M*	*SD*	Social	Quality	Brand	Condition	T responseª
Social	4.33	1.21	1				
Quality	5.03	.88	.52**	1			
Brand	.51	.50	.73**	.47**	1		
Condition	.50	.50	.03	.13	.07	1	
T response^a^	13.82	24.51	− .10	.05	.06	.04	1

ªDifference between post-viewing testosterone level (${T}_{2}$) and baseline testosterone level (${T}_{3}$)

*p < .05. **p < .01

To test ${\mathrm{H}}_{2}$ and ${\mathrm{H}}_{3}$ we conducted a multivariate analysis of variance (ANCOVA) with Condition (Alone, Together), Social, Quality, and the interaction between Condition and the two luxury dimensions (Condition * Social and Condition * Quality) as independent variables and the T-level difference (T response = T sample T2 – T sample T3) as dependent variable. Table 2 reports the results of this analysis.

**Table 2:** Ancova results for participants’ T responses

	*F*-value	*p*-value	η^2^
Condition (Alone/Together)	**6.73**	.011	.08
Quality	1.82	.181	.02
Condition * Quality	**5.93**	.017	.07
Social	2.43	.123	.03
Condition * Social	.04	.846	.00

Significant effects are indicated in bold

**Fig. 2:**
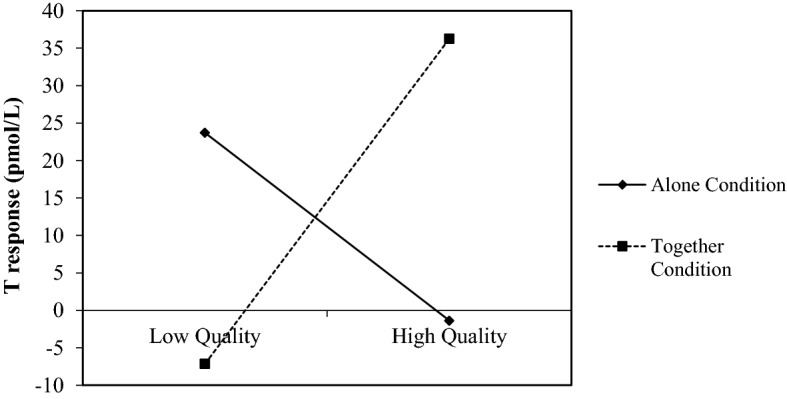
Interactive effect of condition and quality on T Response

While Quality and Social dimensions had no significant effect on participants’ T response (F = 6.73, *p* = 0.181, and F = 1.82, *p* = 0.123, respectively) we did find a significant main effect of Condition (F = 6.73, *p* = 0.011). As hypothesized, the main effect was qualified by a significant interaction effect between Condition and Quality (F = 5.93, *p* = 0.017). However, the interaction effect between Condition and Social was not significant (F = 0.047, *p* = 0.846). Figure 2 shows the direction of the significant interaction effect between Condition and Quality on male participants’ T response. The relationship between Quality and participants’ T response was negative when in the Alone condition and positive in the Together condition. Results of simple slope analyses showed that the relationship between quality and T response was significant and positive for the Together condition (B = 21.70; t = 2.67; *p* =  0.009) and non-significant for the Alone condition (B = − 12.54; t = − 1.66; *p* = 0.102).

## Discussion

This study investigates consumer self-reported and testosterone responses to advertising of luxury branded goods. In line with ${\mathrm{H}}_{1}$, we found that luxury branded goods score higher on social and quality value than non-luxury branded goods. The hormonal results provide no support for ${\mathrm{H}}_{2}$. There was no direct association between post-viewing T levels, social and quality value of the advertised branded goods. Finally, we found partial confirmation of ${\mathrm{H}}_{3}$. The results indicate a positive association between the change in post-viewing T levels and quality value for the branded goods shown in the ads. However, this effect was only present in the Together condition.

### Theoretical implications

Consumers differ in their perceptions of luxury (Dubois & Duquesne, 1993). Some consumers may view a Seat car as a luxury brand, while others may consider it non-luxury (Dubois et al., 2005). In the same line of reasoning, traditional luxurious brands such as Mercedes-Benz and Bentley may both be perceived as luxury, but one compared with the other may be perceived as less luxurious. Thus, an important question in luxury research is to understand how different social and quality motivations relate to the personal and contextual antecedents (Eisend & Schuchert-Güler, 2006).

Our findings contribute to the understanding of consumer responses to advertising of luxury goods by providing for the first time—to the best of our knowledge—a biological correlate for the distinct value of luxury goods. Luxury goods may create different expectations in consumers, such as enabling a gain in social status or providing comfort, both of which are associated with activation of the neurotransmitter testosterone in the brain (Eisenegger et al., 2011). During the stimulus-free period participants were left to express their thoughts and feelings about the advertising in a spontaneous way. Analysis of their conversations revealed that they expressed thoughts and feelings not only about the cars but also about the quality and execution of the ads. For instance, when viewing an ad of a luxury car in a social condition, participants included positive comments: “I like this ad”, “Good TV commercial”. Interestingly, in many cases the participants fantasized about owning the Mercedes-Benz shown in one of the ads: “Compared to the other [cars], it was more about the functions of the car because in the previous [ad] it was just a story line [about the] countryside and now it’s [got] more controllable display [and] adjustable seats. [It] addresses the lifestyle, [like] when you don’t have to drive, [you] want to relax on the back seat [but] if you want to drive yourself then you can still enjoy the power of the car. Also if you had [to go on] a long drive, you [would] get out and still be fresh. It [the Mercedes] covers all the options. I think [it is showing us] the ideal life, or what we are planning to achieve as university students, that’s how we want to see ourselves later on. It [the ad] is really targeted at us. Now we’re choosing the car we will buy in ten years’ time. If I had the money, I’d buy this car.”

It is not surprising that many respondents confess that viewing an advertising of luxury cars without consuming or buying them is a nice pastime because it fulfills their desire to possess these goods, even if only in imagination (Arnold & Reynolds, 2003; Brakus et al., 2009). Plassmann and Wager (2014) label this process the ‘placebo effect of luxury goods on the consumer’, meaning that luxury goods are expected to have higher quality or are expected to provide consumers with social benefits, which might evoke dreams of how others might regard them if they obtained the associated high status. The abovementioned analysis of the respondents’ conversations illustrates that viewing luxury-goods advertising can be a rewarding experience which can help consumers better assess the social and quality value of the advertised goods and testosterone seems to play an important role in this process.

Reward processing, which plays a key role in social interactions and social hierarchies has frequently been related to testosterone (Sanfey, 2007). Previous studies report evidence of a connection between reward-sensitive brain areas and T levels suggesting that an increase in T levels can indeed be defined as rewarding (Eisenegger et al., 2011). Neuroscience studies also provide evidence of the association between the brain reward system and observing luxury branded goods (Plassmann et al., 2012). Thus, one explanation for our findings could be that enhanced T levels during an ad viewing of luxury cars indicates a rewarding experience for consumers who would attain a material benefit from consuming luxury branded goods. For instance, enhanced T levels in response to viewing an ad of Mercedes-Benz can be rewarding for consumers who perceive this branded car as scoring high on quality (e.g. comfort, security, interior finishes). Importantly, value perception can occur without actual consumption or usage of the product, which is exactly what happened to our participants (Woodruff, 1997). Luxury goods can fulfill material and social expectations in the pre-purchase stage (i.e. ad viewing). Viewing an advertising of a luxury car can be a rewarding experience in the same way as recalling a previous victory (Carré & Putnam, 2010), watching a soccer game (Van der Meij et al., 2012) or looking at a cute face (Hahn et al., 2015). For instance, some participants stated that just viewing the ad of the luxury car was a rewarding experience in itself: “Emotional experience, okay. I liked the car. I liked the product and also liked the atmosphere around it.” Or “The Porsche impressed me. Speed and sound. I’d be really glad to own it. I’d be really proud.”

Another important finding of this study is that social context influences the association between the perceived value of luxury goods and the related hormonal response. Previous research suggests that watching sporting events alone or with a group modulates gratification (Harris & Sanborn, 2013). Specifically, watching sports in a group rather than alone is a more enjoyable, emotionally involving and rewarding experience (Harris & Sanborn, 2013). Thus, viewing an ad of luxury goods and/or sharing your thoughts about an ad of luxury goods with other people might amplify this rewarding experience in the same way as it does when watching sports together with other people (e.g. in the stadium, in a bar, or at home with friends), (Pozharliev et al., 2017).

Past studies found that merely observing a similar or successful other person increases consumers’ expectations about their own future wealth, which in turn increases their desire for luxury goods (Mandel et al., 2006). Thus, another explanation for our findings could be that the increase in T levels while viewing luxury-goods advertising reflects activation of human social competitiveness in consumers who perceive quality as an indicator of competitive success. This second explanation resonates with Carré and Putnam (2010) who found that watching a previous victory enhances T levels, whereas watching a neutral video had no effect. In addition, viewing advertising of luxury goods or talking about luxury branded goods with another person could partially recreate or resemble a socially competitive situation, which explains the increase in T levels in the Together compared to the Alone condition. One participant described the Range Rover as “more functional. I think a bit of both, stylish, focused on details, just in every stitch of the leather. Appeals to a more serious type of person. Inside, you see a wooden style in the screens. It’s more like a status car, more for a consultancy CEO. Not an economical car.” Meanwhile, the Mercedes-Benz is regarded as: “The HD things and the way they show the details is more functional than the last one because it has descriptions. [It’s] really focused on the inside, focused on somebody really important who has his own driver and can use his laptop [on the go]. He’s an important guy, with champagne and message seats, successful. A rich guy with a big house.”

Our findings resonate with previous research that looks at individual differences in luxury value perception (Shukla & Purani, 2012; Wiedmann et al., 2007). Some consumers derive more utility from quality and functional characteristics of luxury goods while others value the ability of the luxury good to make an impression on others. In a recent cross-cultural study on perception of luxury value dimensions Hennigs et al. (2012) found that consumers in various parts of the world perceive luxury value differently and prefer luxury goods for different reasons. Authors identify four distinct clusters of consumers: luxury lovers, status-seeking hedonists, the satisfied unpretentious [user] and the rational functionalist. For instance, US, Indian and Japanese consumers (status-seeking hedonists) perceive the social dimension of luxury goods to be extremely important “*I like to own things that impress people*” (Hennigs et al. 2012, p.1029). In relation to the functional dimension of luxury value perception, German consumers (rational functionalists) compared to the consumers in other countries value quality and performance characteristics of the luxury good more. Germans show the highest mean ratings on functional value: “*I place emphasis on quality assurance over prestige when considering the purchase of a luxury brand*” (Hennigs et al., 2012, p.1025).

Note that almost 80% of our participants were either Dutch or German, which may explain why we found no association between post-viewing T levels and social utility of luxury cars, neither in terms of a main effect nor in terms of an interaction effect with the social condition (Together/Alone). According to Hennigs et al. (2012), Germans derive pleasure from and place emphasis on quality, performance and substantive attributes rather than on the social value of luxury goods, which may explain why we found a positive association between post-viewing T levels and quality utility for the branded cars shown in the ads. A survey conducted in Denmark, reported that 48% of BMW owners buy luxury branded goods for personal reasons (e.g. quality and performance) rather than for socially driven reasons (Tsai, 2005). Of course, this cross-cultural interpretation of our results assumes that Dutch male consumers value quality and performance characteristics more and place less emphasis on the social utility of luxury cars. Cross-cultural research by Project GLOBE found that Germany and the Netherlands fall into the same cultural cluster (‘Germanic cluster’) and are thus relatively similar to each other in terms of cultural values (House et al., 2004). Thus, we assume that Dutch male consumers are more similar to the neighboring German and Danish consumers rather than to Indian, Japanese, Brazilian or American consumers. When expressing their thoughts about cars (luxury and non-luxury) our participants used more quality-related words or phrases than social words or socially related phrases. Importantly, when viewing the ad of the luxury cars in the social condition almost all quality words and quality phrases that our participants used were positive such as: “fast car, smooth design, great engine noise, very high quality product, driving comfort, high quality car, enjoy the power of the car, it was really sophisticated with a lot of attention to the details, I want to buy the car, it looks great, it’s sophisticated, drives f… fast and the sound is great.”

On the other hand, when viewing the ad of non-luxury car in the social condition, participants still used more quality than social words and phrases but in this case they were slightly more negative than positive, such as: “I think in the first one we saw too many features; it doesn’t really give me the feeling I want to drive the car; it’s really boring.” and “For such an ugly car, why they focused so much on the esthetics and the way it looks [is beyond me]. The whole time you’re looking at shapes and lights.”

The type of the luxury goods used in this study also resonates with the abovementioned reasoning. Although the social dimension still plays an important role, the quality dimension could have a larger impact on consumers’ appraisal and preference for a car, especially compared to products such as sunglasses and handbags, which offer a limited number of distinctive physical attributes and for which the differences in terms of quality are less visible. Quality of the internal materials, acceleration, comfort, design, extras, and security are just some of the physical attributes that shape the perceived brand value of a car. For instance, for some consumers, the superior expected brand utility of Ferrari could derive from the unparalleled sound of its exhaust. Porsche fans, on the other hand, might attribute more value to the distinctive external design of the car. Even without the emblem, a Porsche is still easily recognizable as a Porsche; it will not be mistaken for another brand (Han et al. 2010). In contrast, a Mercedes-Benz without the star on its hood could easily be confused with a SsangYong (Web Appendix C). While the external appearance of a luxury good and the social benefits it brings can be partially replicated (e.g., Louis Vuitton bags counterfeited in the Asian market), the quality value of a luxury good is more difficult to counterfeit. The SsangYong Chairman looks much like a Mercedes-Benz on the outside but the quality, comfort, security and interior of the S-class Mercedes are superior. In sum, the nature of the luxury goods used in this study might explain why quality and not social value generates a rewarding experience, reflected by salivary testosterone and moderated by social context.

### Practical implications

Our research might also offer practical implications for marketing communication managers looking for new ways to enhance the impact of their marketing communication, and in particular for those managers interested in empirical evidence supporting their decision to invest in luxury-goods advertising. First, regardless of the level of perceived quality and/or social value of the luxury good, marketers should try to create social platforms on which consumers can experience the luxury-goods advertising intensely. Social context is likely to enhance consumer responses to luxury-goods advertising (Pozharliev et al., 2017). In online settings, in might be appropriate to motivate consumers to view marketing communication in social context (e.g. stadiums, shopping malls, concerts), so that the presence of others enhances their responses to the advertised goods.

Second, advertising of luxury goods should leverage not only the social, but also the quality value of the advertised products. Our results provide also insights into markets where the focus of the luxury-goods advertising could be more on the quality vs. social value of the brands (e.g. the Netherlands, Germany, Scandinavian countries). Specifically, our results indicate that there is a strong association between hormonal responses to viewing of advertising of luxury branded goods and the way male consumers appraise them on material but not on social value. The association between T levels and quality value of luxury branded goods can explain the strong appreciation and desire of certain consumers for original luxury branded goods even in the presence of counterfeited branded goods (e.g., Gucci hats counterfeited in Asian markets) and products mimicking luxury goods (e.g., cheaply priced chic goods). Indeed, these consumers are willing to pay a premium price for original luxury goods even when most consumers find it difficult to distinguish between a real and counterfeit luxury good simply by observing it from a distance (i.e., high resemblance in terms of product form). Consumers’ preference for original luxury goods prevails when the social value does not imply individual rewarding benefits and when the material value of the luxury good (i.e. quality, reliability, craftsmanship) cannot be easily replicated by counterfeit goods. Our findings resonate with previous research on counterfeit luxury brands. Wilcox et al., (2009) found that consumers’ desire for counterfeit luxury brands depends on social motivation factors (e.g. gaining approval in social situation). In contrast, when consumers hold a value-expressive attitude (e.g. self-expression) toward the luxury brand, factors such as quality and reliability (i.e., functional appeal of cars) influence their preferences and value perception.

Finally, our study clearly indicates that managers should use a mixed set of market research tools to investigate consumer responses to advertising of luxury goods (Venkatraman et al. 2015). Specifically, our results show that changes in T levels affect consumer responses to luxury-goods advertising and thus offer a complementary to traditional marketing methods insights into consumer experiences with luxury goods (Bagozzi & Lee, 2019). Therefore, marketers should incorporate consumer neuroscience methods to better understand and predict consumer responses to their marketing communication (Pozharliev et al., 2017).

### Limitations and further research

First, the majority of our respondents were Dutch and German males. Previous research indicates that consumers in various parts of the world differ significantly in the way they respond to luxury goods (Hennigs et al., 2012). Some consumers derive satisfaction from the functional value of luxury goods while others like things that impress people (Hennigs et al., 2012). Thus, future research should look at cross-national variations in the associations between the change in testosterone levels and consumer responses to advertising of luxury goods. For instance, consumers in developing countries (e.g. India, Russia) who place emphasis on the social value of luxury may display different hormonal patterns compared to consumers in developed countries (Shukla & Purani, 2012).

Second, individual differences such as personality traits could influence consumers’ hormonal responses to luxury-goods advertising. For instance, people’s personality traits affect testosterone levels. People with a high need for power have higher testosterone levels on encountering a challenger or merely being primed with a status cue (Schultheiss et al., 1999). Another study found a negative association between testosterone and trust in socially naïve humans (Bos et al., 2010). These authors suggested that testosterone increases social vigilance in naïve humans to better prepare them for competition over status and valued resources. Moreover, our study did not take into account the level of interpersonal closeness (e.g. friends versus strangers) between participants in the Together condition. A recent study has linked testosterone levels and interpersonal closeness (Ketay et al., 2017). Specifically, the authors found a negative correlation between testosterone levels and desired closeness between recently acquainted strangers (Ketay et al., 2017). Future research could explore whether the level of interpersonal closeness between subjects influences the way they appraise luxury branded goods in a social context as compared to being alone, and whether possible variations in perceived utility of luxury goods can be associated with changes in testosterone levels.

Third, our research included only ads showing luxury versus non-luxury cars. Future research should examine consumers’ testosterone responses to different types of luxury goods (e.g. original luxury watches versus counterfeit luxury watches). For instance, consumers’ hormonal responses to products with limited physical features, such as hats and shoes where the difference in terms of quality between the original and the counterfeit product is less visible, could differ from consumers’ hormonal responses to cars or other goods for which the quality characteristics are difficult to imitate.

Fourth, our results are based on the testosterone responses of men in their 20 s and 30 s who are in the prime of their lives and whose hormonal levels are at their peak. Previous research suggests that age significantly affects circulating testosterone concentrations (Vingren et al., 2010). Aging beyond 40 years is associated with a 1–3% decline per year in circulating testosterone concentration in men. Future research could explore whether consumer testosterone responses to advertising of luxury goods will differ across different age groups.

Finally, previous research suggests that gender plays an important role in hormonal production and regulation (Sapienza et al., 2009). This study focuses on consumer testosterone responses of male participants because previous research has suggested that the relationship between (competitive) social status and testosterone may be more prominent in males than females (Carré & Putnam, 2010; Saad & Vongas, 2009). However, females are equally interesting to study. Thus, the relationship between social status, luxury goods and hormones in females is an interesting research question that deserves more attention. For instance, the female response to luxury goods in various social contexts may be associated with physiological and biological mechanisms (e.g. hormones) different than those for males.

## Supplementary Information

Below is the link to the electronic supplementary material.

Supplementary file1 (DOCX 28 kb)

Supplementary file2 (DOCX 31 kb)

Supplementary file3 (DOCX 196 kb)
