# Supplementary material for: Consumer self-reported and testosterone responses to advertising of luxury goods in social context
Source: Ital. J. Mark. 2021 Apr 21;2021(1-2):103–27. doi: 10.1007/s43039-021-00023-y (PMC8059690; doi:10.1007/s43039-021-00023-y)
Supplement: Supplementary file 1 — Supplementary file1 (DOCX 28 kb) [file 43039_2021_23_MOESM1_ESM.docx]

**Web Appendix A:**

LIST OF ADVERTISMENTS OF LUXURY AND NON-LUXURY CARS (PER BRAND AND MODEL) DISPLAYED DURING THE EXPERIMENT

| LUXURY BRAND | LUXURY MODEL | NON-LUXURY BRAND | NON-LUXURY MODEL |
| --- | --- | --- | --- |
| BMW | I8/SPORT | RENAULT | DUSTER/SUV |
| ROLLS ROYCE | DAWN/SPORT | KIA | SPORTAGE/SUV |
| RANGE ROVER | AUTOBIOGRAPHY/SUV | OPEL | MOKKA/SUV |
| FERRARI | F430/SPORT | SEAT | LEAN/ST |
| MERCEDES-BENZ | S-CLASS/LIMOUSINE | SKODA | SUPERB/LIMOUSINE |
| ASTON MARTIN | DBS/SPORT | FIAT | AEGEA/SEDAN |
| PORSCHE | CAYENNE/SUV | SUBARO | LEGACY/SEDAN |
