# Supplementary material for: Consumer self-reported and testosterone responses to advertising of luxury goods in social context
Source: Ital. J. Mark. 2021 Apr 21;2021(1-2):103–27. doi: 10.1007/s43039-021-00023-y (PMC8059690; doi:10.1007/s43039-021-00023-y)

**Web Appendix B:**

Use this scale to answer the following questions:

1. Strongly disagree
2. Disagree
3. Disagree somewhat
4. Undecided
5. Agree somewhat
6. Agree
7. Strongly agree
8. This brand has consistent quality.


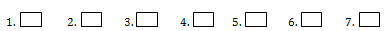


1. This brand is well made.


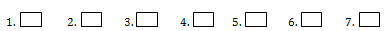


1. This brand has an acceptable standard of quality.


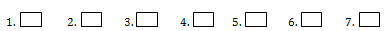


1.
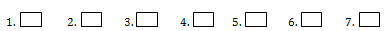
This brand has poor workmanship.

1. This brand would not last a long time.


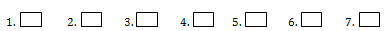


1. This brand will perform consistently.


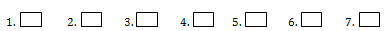


1.
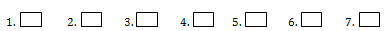
This brand would help me to feel acceptable.
2. This brand would improve the way I am perceived.


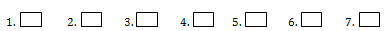


1. This brand would make a good impression on other people.


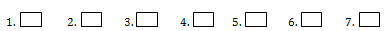


1. This brand would give its owner social approval.


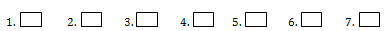

Supplement: Supplementary file 2 — Supplementary file2 (DOCX 31 kb) [file 43039_2021_23_MOESM2_ESM.docx]
