# Supplementary material for: Consumer self-reported and testosterone responses to advertising of luxury goods in social context
Source: Ital. J. Mark. 2021 Apr 21;2021(1-2):103–27. doi: 10.1007/s43039-021-00023-y (PMC8059690; doi:10.1007/s43039-021-00023-y)
Supplement: Supplementary file 3 — Supplementary file3 (DOCX 196 kb) [file 43039_2021_23_MOESM3_ESM.docx]

**Web Appendix C:**

EXTERNAL DESIGN SIMILARITIES BETWEEN
MERCEDES-BENZ S-CLASS (LEFT) AND SSANGYONG CHAIRMAN

**
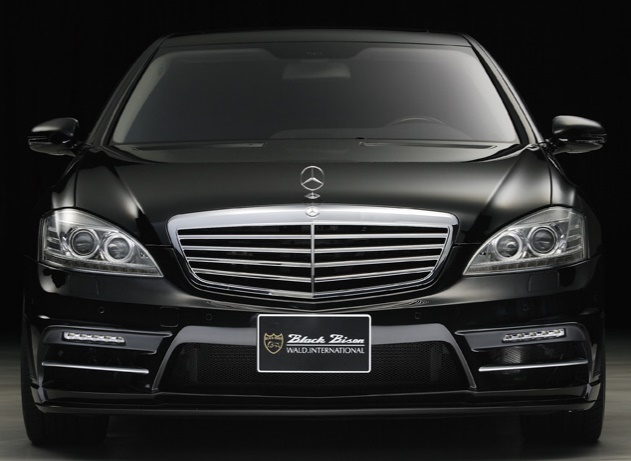

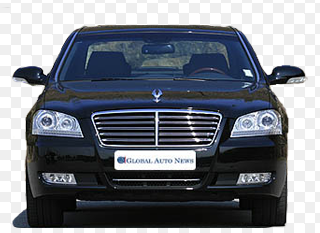
**
